# Evaluating Craniofacial Morphology Ratios as Predictors of Obstructive Sleep Apnea Severity in Non-Obese Adult Males

**DOI:** 10.3390/dj12120374

**Published:** 2024-11-21

**Authors:** Masasuke Shimatsu, Shigeto Kawashima, Mitsuyoshi Suzuki

**Affiliations:** 1 Isdorly Orthodontic Office, Tokyo 101-0047, Japan; masasuke@zb4.so-net.ne.jp (M.S.); isdorly1187@gmail.com (S.K.); 2 Department of Pediatrics, Faculty of Medicine, Juntendo University, Tokyo 113-8421, Japan

**Keywords:** obstructive sleep apnea, craniofacial morphology, cephalometric analysis, apnea–hypopnea index

## Abstract

**Background**: This study aimed to examine the connection between craniofacial morphology, particularly the horizontal and vertical dimensions of the mandible, and the severity of obstructive sleep apnea (OSA) in non-obese adult males by utilizing a cephalometric analysis and introducing a new skeletal ratio index. **Methods**: A cohort of 44 non-obese adult males with OSA, diagnosed via the apnea–hypopnea index (AHI) from polysomnographic recordings, was evaluated using a lateral cephalometric analysis. OSA severity was classified as mild (5 ≤ AHI < 15) in 19 patients, moderate (15 ≤ AHI < 30) in 15 patients, and severe (AHI ≥ 30) in 10 patients. The S-Go distance divided by the N-Me distance (S-Go/N-Me) was used as a vertical ratio of craniofacial morphology, the Go-Me distance divided by the S-N distance (Go-Me/S-N) was used as a horizontal ratio, and the results were compared between groups. Correlations between each ratio and craniofacial morphology based on the five factors from the Ricketts analysis were examined for each group. **Results**: A significant difference was found in the horizontal ratio Go-Me/S-N between the mild and moderate groups (*p* < 0.05) and the mild and severe groups (*p* < 0.05). However, no significant differences in Ricketts analysis factors were observed across OSA severity groups. Correlations between the Go-Me/S-N and Ricketts factors were identified in the mild and moderate groups but not in the severe group. The horizontal skeletal dimension Go-Me/S-N was strongly associated with OSA severity. **Conclusions**: The horizontal mandibular ratio Go-Me/S-N, independent of body shape, may offer a valuable morphological marker for differentiating OSA severity in non-obese males.

## 1. Introduction

Obstructive sleep apnea (OSA) is a prevalent sleep disorder characterized by repetitive episodes of upper airway obstruction during sleep, leading to the reduced or complete cessation of airflow despite ongoing respiratory efforts. This condition is associated with significant morbidity, including cardiovascular disease, cognitive impairment, and reduced quality of life [1,2,3,4]. The severity of OSA is commonly quantified using the apnea–hypopnea index (AHI), which measures the frequency of apneic and hypopneic events per hour of sleep [5].

Craniofacial morphology has been identified as a significant contributor to the development of OSA, particularly in non-obese populations. While obesity is a predominant risk factor for OSA in Western populations, abnormalities of the maxillofacial skeleton, including reduced mandibular dimensions and retrognathia, are more commonly implicated in Asian populations [6]. These skeletal characteristics can predispose individuals to airway narrowing and subsequent obstruction during sleep. Structural factors of the maxillofacial skeleton have been identified as the factors most significantly related to the severity of OSA among Japanese patients with OSA [7]. Mandibular retroversion, in which the mandibular dentition is positioned posteriorly compared to the maxillary dentition during occlusion, is a structural abnormality of the maxillofacial skeleton associated with mandibular growth. In such a skeleton, the tongue is positioned more posteriorly than normal, and when muscular activity is reduced during sleep, the root of the tongue can easily obstruct the airway and cause apnea [8,9].

Previous studies investigating the relationship between craniofacial morphology and OSA severity have produced mixed results. Some research has suggested that reduced mandibular size exacerbates airway obstruction, thereby increasing OSA severity [10,11,12,13]. Conversely, other studies have found no significant correlation between mandibular dimensions and the severity of OSA, suggesting that additional factors may be involved in the pathophysiology of this disorder [14]. Patients with severe OSA who are not obese tend to have a short face and deep overbite rather than a dolichofacial pattern (long face), according to the Ricketts analysis [15,16,17]. However, the size of the mandible does not necessarily affect the severity of OSA. In the horizontal skeletal morphology of OSA patients, the relationship between the AHI and mandibular length, in particular, has not been clarified. To date, no research has concentrated on the vertical and horizontal ratios of jaw and facial morphology instead of relying on measurements that depend on body shape.

This study aimed to clarify the relationship between craniofacial morphology, specifically the horizontal and vertical skeletal dimensions of the mandible, and the severity of OSA in a cohort of non-obese adult males. By employing a cephalometric analysis and new ratio-based skeletal indices, this research sought to provide a more nuanced understanding of how mandibular dimensions impact OSA severity and to identify reliable morphological markers for differential diagnosis and treatment planning.

## 2. Materials and Methods

### 2.1. Patients

The patients were selected based on their OSA symptoms, such as heavy snoring and witnessed apneic episodes at Juntendo University Sleep Laboratory (Bunkyo, Japan) and our private sleep clinic in Tokyo between April 2016 and March 2017. The subjects were 44 non-obese adult males who had never received any intervention, such as continuous positive airway pressure or mouthpiece therapy at night, with OSA and body mass indexes of 20.0–25.0 kg/m^2^. Before the consultation, none of the patients had any history of orthodontic treatment or facial skeletal surgery, such as uvulopalatopharyngoplasty or maxillomandibular advancement. The information used was the AHI and lateral cephalograms. The AHI diagnosed OSA from all-night polysomnographic recordings taken at the private sleep clinic and Juntendo University Sleep Laboratory. The severity classifications followed the classification system widely used by the Society of Sleep Research [18], with 19 patients in the mild group (5 ≤ AHI < 15), 15 in the moderate group (15 ≤ AHI < 30), and 10 in the severe group (AHI ≥ 30) (Table 1). The study followed the Declaration of Helsinki and was approved by the Institutional Review Board of Juntendo University (approval nos. 15–219, 17 March 2016). The participants were enrolled in the study under an opt-out consent model, whereby they were included unless they explicitly declined to participate.

### 2.2. Cephalometric Analysis

**Figure 1:**
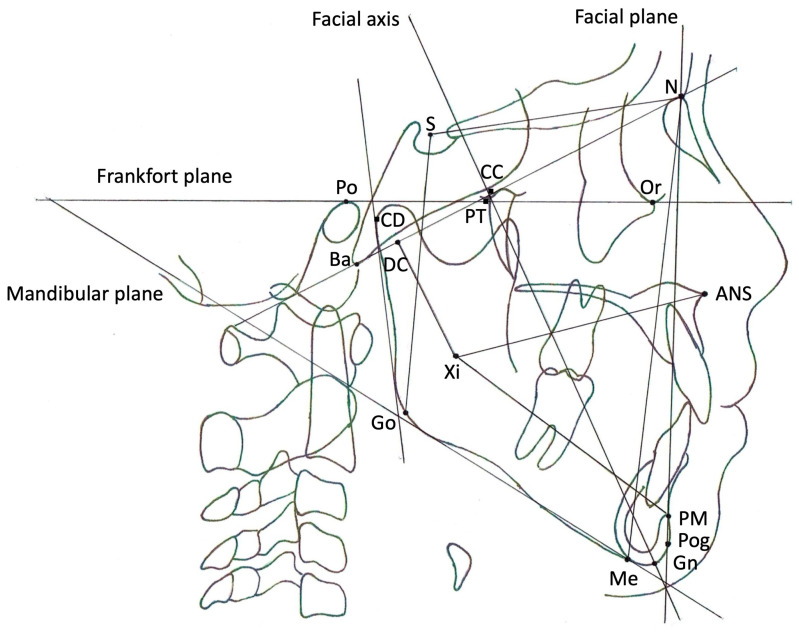
Skeletal reference points and lines on lateral cephalometric radiographs. Reference points: N—the most anterior point of the frontonasal suture; S—the central point of the pituitary fossa (sella) of the sphenoid bone; Or—the most inferior point on the lower border of the bony orbit; Po—the most superiorly positioned point of the bony external auditory canal; ANS—the most anterior point of the bony nasal floor; PM—the point at which the curvature of the anterior border of the symphysis changes from concave to convex; Pog—the most prominent point of the chin; Gn—the point on the chin determined by bisecting the angle formed by N-Pog and the mandibular plane; Me—the most inferior point of the symphysis; Go—the point on the curvature of the angle of the mandible located by bisecting the angle formed by the tangents to the posterior ramus and the inferior border of the mandible; Ba—the most inferoposterior point of the occipital bone at the anterior margin of the occipital foramen; CC—the point at which Ba-N and CC-Gn intersect; DC—a point selected in the center of the neck of the condyle on the Ba-N; Xi—the point located at the geographic center of the ramus; PT—the intersection point of the inferior border of the foramen rotundum with the posterior wall of the pterygomaxillary fissure; CD—the most posterior superior point on the condyle of the mandible. Reference lines: facial axis—the line connecting CC and Gn; facial plane—the line connecting N and Pog; mandibular plane—the tangent to the inferior border of the body of the mandible that passes through Me; Frankfort plane—the line connecting Po and Or.

Figure 1 shows the cephalometric measurements used in this study. Lateral cephalometric radiographs were taken with the subject sitting upright in a chair at our clinic to meet the requirement of having the Frankfort horizontal line (FH) parallel to the floor. Cephalometric measurements related to craniofacial morphology were measured using WinCeph software (ver11, Rise Co., Miyagi, Japan) to trace lateral cephalograms. Two independent orthodontists measured the values, and the average of those two measurements was adopted. Skeletal reference points are shown in the footnote of Figure 1. Vertical or horizontal measurement references were defined as follows: S-Go—the distance between S and Go (mm); N-Me—the distance between N and Me (mm); S-Go/N-Me—the S-Go distance divided by the N-Me distance (ratio, %); Go-Me—the distance between Go and Me (mm); S-N—the distance between S and N (mm); and Go-Me/S-N—the Go-Me distance divided by the S-N distance (ratio, %). Five factors derived from the Ricketts analysis were measured in each subject. The following are the definitions of these five factors: facial axis (FA)—the angle between PT-GN and BA-N (degrees); facial depth (FD)—the angle between N-Pog and Po-Or (degrees); mandibular plane angle (MPA)—the angle between Po-Or and Me-Go (degrees); lower facial height (LFH)—the angle between ANS-XI and XI-PM (degrees); and mandibular arc (MA)—the angle between XI-PM and XI-CD (degrees).

### 2.3. Statistical Analysis

All statistical analyses were performed using the SPSS statistical software package (Windows version 11.0; SPSS Japan, Tokyo, Japan). The tests were one-tailed, and values of *p* < 0.05 were considered significant. The Kruskal–Wallis test was used to determine significant differences among three groups, whereas the Mann–Whitney U test was used for comparisons among two groups. Spearman’s rank correlation analyses assessed the significance of the relationships between vertical or horizontal ratios and the five factors from the Ricketts analysis.

## 3. Results

### 3.1. Relationship Between Ricketts Analysis and Maxillofacial Morphology in OSA Patients

**Figure 2:**
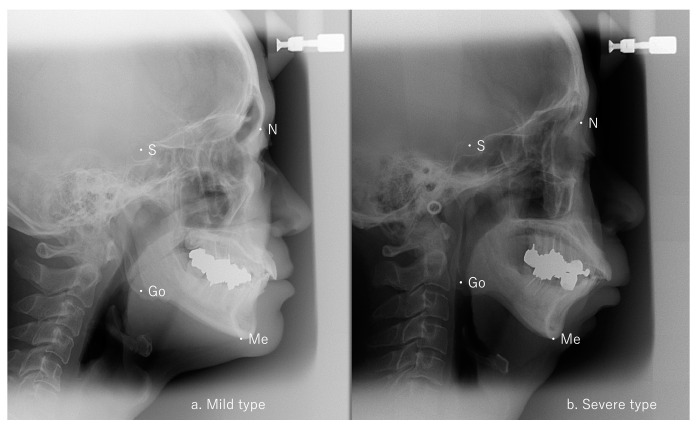
Lateral cephalometric radiographs. (**a**) Mild AHI patient: S-Go/N-Me 65.6% and Go-Me/S-N 102.1%, (**b**) severe AHI patient: S-Go/N-Me 67.5% and Go-Me/S-N 99.8%, AHI: apnea–hypopnea index.

A significant difference in Go-Me/S-N was identified between the mild and moderate groups (*p* < 0.05) and between the mild and severe groups (*p* < 0.05) but not between the moderate and severe groups (Table 1). Representative cephalometric analyses of the mild and severe groups are shown in Figure 2. No differences among the three groups were seen for any of the five factors from the Ricketts analysis (Table 1).

### 3.2. Relationship Between Ricketts Factors and Go-Me/S-N

Correlations between the horizontal ratio of maxillofacial morphology Go-Me/S-N and factors from the Ricketts analysis were found in the mild and moderate groups for facial depth, but not in the severe group (Table 2).

## 4. Discussion

The Ricketts analysis, a form of cephalometric analysis, is widely used to assess the craniofacial morphology of OSA patients. The analysis includes the five factors of FA, FD, MPA, LFH, and MA to indicate craniofacial morphology and is frequently applied in cephalometric studies [17,19,20,21]. Kikuchi et al. compared cephalograms between 29 children (mean age, 6.3 years) with hypertrophied tonsils and adenoids, loud snoring, and sleep apnea, and 41 controls (mean age, 9.0 years) and reported significant differences in FA, LFH, and MA between the groups [21]. Similarly, Higurashi et al. examined 44 Japanese adult OSA patients (AHI > 10; mean AHI, 56.0 ± 25.2; 41 males, 3 females; aged 44.6 ± 13.0 years) and 34 controls (AHI = 0; 7 males, 27 females; aged 26.1 ± 8.7 years) using the Ricketts and Downs–Northwestern analyses [20]. They found significant differences between the OSA and control groups in FA and LFH when using the Ricketts analysis, but no significant differences with the Downs–Northwestern analysis. These findings suggest that the Ricketts analysis is effective in analyzing the craniofacial morphology of OSA patients.

The vertical craniofacial morphology of OSA patients is often characterized by a dolichofacial pattern (long face) [15,16,17,20,21]. However, non-obese patients with severe OSA have been reported to have a short LFH, or a short face with a deep overbite [19]. Previous studies have found no significant relationship between horizontal skeletal factors, particularly mandibular length, and the AHI severity classification in OSA patients. Similarly, no association has been observed between the AHI and vertical skeletal factors such as N-Me and S-Go. This study examined the relationship between vertical and horizontal skeletal factors in OSA patients across various sexes and ages. Specifically, we measured vertical components such as S-Go and N-Me, and horizontal components including Go-Me, SN, and Xi-PM (corpus length), which are commonly used in cephalometric analysis. However, no association was identified between the vertical ratio S-Go/N-Me and OSA severity as reflected by the AHI in our study of non-obese adult male OSA patients. No significant differences were observed across the three OSA severity groups in the five Ricketts analysis factors. Of the five Ricketts factors, only FD (representing the anteroposterior positional relationship of the jawbone) showed a relationship in the mild and moderate groups, although the correlation was weak. No correlation was found for FA, another factor representing the anteroposterior positional relationship of the jaw.

To address this, in addition to the vertical ratio S-Go/N-Me, the horizontal ratio Go-Me/S-N was introduced as a new skeletal factor value independent of body shape. Our analysis revealed that the horizontal skeletal factor was associated with OSA severity. Specifically, the craniofacial horizontal ratio Go-Me/SN differed significantly among mild, moderate, and severe OSA groups. Pairwise comparisons showed differences in horizontal ratio Go-Me/SN between the mild and moderate groups and between the mild and severe groups. As the AHI severity increased, the horizontal ratio decreased. This study introduces a novel approach to understanding the correlation between mandibular length and OSA severity by using ratio values instead of traditional measurements. We propose that the horizontal ratio Go-Me/SN may serve as a valuable index for distinguishing between mild, moderate, and severe OSA. The horizontal ratio could be a helpful index for evaluating OSA severity in clinical settings, especially for non-obese individuals. This metric could simplify evaluations and potentially improve patient management strategies.

The study has limitations due to its small sample size of 44 non-obese adult male patients, limiting generalizability. In addition, the absence of female participants hinders the understanding of potential sex-related differences in OSA severity and craniofacial morphology. Further, the study lacked a control group of healthy individuals without OSA, making the differentiation of OSA-related craniofacial characteristics difficult. In addition, the study design was observational, so while it identified an association between the horizontal Go-Me/S-N ratio and OSA severity, it did not establish causation. Further research would be needed to determine whether changes in this craniofacial dimension directly contribute to OSA severity or are merely correlated with it. A more extensive and diverse population, including females and controls, is needed to clarify these findings.

## 5. Conclusions

A novel horizontal ratio Go-Me/S-N correlates with OSA severity, distinguishing between mild, moderate, and severe cases. This finding suggests that horizontal skeletal ratios can serve as reliable markers in assessing OSA severity, offering an alternative to conventional craniofacial measurements and potentially simplifying clinical evaluations for non-obese patients.

## Figures and Tables

**Table 1:** Patient demographic and cephalometric characteristics.

OSA Severity	Mild	Moderate	Severe	*p*-Value
Number of patients	19	15	10	
Age (years)	45.0 (38.0–52.5)	47.0 (41.5–59.5)	51.0 (46.3–60.3)	N.S.
AHI (events/h)	10.0 (8.0–11.5)	24.0 (19.4–25.8) **	53.5 (44.5–55.5) **^, ##^	<0.01
Evaluation of maxillofacial morphology				
S-Go/N-Me (%)	67.3 (64.6–70.8)	66.4 (65.4–69.0)	66.9 (65.3–68.4)	N.S.
Go-Me/S-N (%)	109.4 (106.3–114.3)	105.9 (102.3–109.8) *	105.6 (97.1–109.9) *	<0.05
Five factors of Ricketts analysis				
FA (°)	87.3 (84.0–88.7)	87.2 (83.7–89.0)	85.2 (83.3–89.3)	N.S.
FD-FA (°)	88.2 (85.3–89.9)	86.2 (85.0–88.8)	85.2 (83.3–89.3)	N.S.
MPA-FA (°)	24.2 (21.3–29.6)	28.9 (23.4–30.5)	27.0 (23.4–28.4)	N.S.
LFH (°)	50.1 (46.4–51.0)	49.3 (44.6–53.0)	49.5 (48.8–51.6)	N.S.
MA-FA (°)	33.2 (30.6–38.6)	32.0 (28.8–37.8)	32.6 (31.3–34.0)	N.S.

Data are presented as median and interquartile range. Obstructive sleep apnea severity was defined as mild (5 ≤ AHI < 15), moderate (15 ≤ AHI < 30), or severe (AHI ≥ 30). N.S.: not significant. Abbreviations used in this table are available in Figure 1. * *p* < 0.05, ** *p* < 0.01 vs. mild group, ^##^ *p* < 0.01 vs. moderate group.

**Table 2:** Relationship between Ricketts factors and Go-Me/S-N.

Classification		Mild	Moderate	Severe
		(n = 19)	(n = 15)	(n = 10)
FA (°)	r	0.216	0.464	0.401
	*p*	0.360	0.082	0.229
FD-FA (°)	r	0.446	0.593	0.527
	*p*	0.059	0.026 *	0.114
MPA-FA (°)	r	0.172	−0.436	−0.12
	*p*	0.466	0.103	0.729
LFH (°)	r	0.24	−0.115	−0.067
	*p*	0.315	0.989	0.842
MA-FA (°)	r	0.052	−0.071	0.430
	*p*	0.826	0.789	0.197

Facial axis (FA): angle between PT-GN and cranial baseline (from Ba to N); facial depth (FD): angle between facial line (from N to POG) and Frankfort horizontal line (FH; from OR to PO); mandibular plane angle (MPA): angle between FH and mandibular line (from ME to GO); lower facial height (LFH): angle between ANS-XI and XI-PM; mandibular arc (MA): angle between XI-PM and XI-CD. Abbreviations used in this table are available in Figure 1. * *p* < 0.05.

## Data Availability

The data supporting the findings from this study are available from the corresponding author upon reasonable request.
